# A retrospective study concerning specific therapy and evolution of invasive fungal infections diagnosed in the National Institute for Infectious Diseases "Prof. Dr. Matei Balş"

**DOI:** 10.1186/1471-2334-14-S7-P4

**Published:** 2014-10-15

**Authors:** Ramona Zamfir, Olga Dorobăț, Daniela Tălăpan, Roxana Dumitriu, Alexandru Rafila, Elisabeta Benea

**Affiliations:** 1 National Institute for Infectious Diseases "Prof. Dr. Matei Balş", Bucharest, Romania; 2 Carol Davila University of Medicine and Pharmacy, Bucharest, Romania

## Background

Invasive fungal infections still have high morbidity and mortality rates, especially in immunocompromised patients, given the lack of specific symptomatology and of fast and early diagnosis methods.

## Methods

We present a retrospective study performed between January 2011 – June 2014 in the National Institute of Infectious Diseases "Prof. Dr. Matei Balş", including patients with invasive fungal infections and complete clinical and biological data.

## Results

18 patients met the inclusion criteria for the studied period, representing 40% of the patients with potentially invasive fungal infections. The majority of patients were male, and the average age was 44 years. Only 2 of the patients apparently were not immunocompromised, the other 16 presenting HIV infection or fungal infections risk factors. The fungal species identified were *Cryptococcus neoformans* in 8 cases (40%) and *Candida* spp in 12 cases (60%), out of which 75% consisted of non-*albicans* species. The invasive fungi were isolated from blood cultures in 9 cases (52.4%), cerebrospinal fluid in 8 cases (38.1%), tips of central venous catheters in one case and from other pathological products in one case. The average period from admission to identification was 9.6 days. The antifungal susceptibility test indicated that 16 out of the 20 cases (80%) were sensitive to fluconazole and only 20% were dose-dependent sensitivity types. The patients were treated mainly using monotherapy – 1 antifungal in 10 cases (55%). Fluconazole was the most used agent, in 14 cases (77.8%) followed by voriconazole in 7 cases and posaconazole in 4 cases. The average treatment duration was 37.7 days, the shortest being one day, and the longest 120 days. 6 out of 18 patients (33.3%) deceased: one patient presenting severe bacterial infection treated with prolonged antibiotherapy and 5 presenting *C. neoformans* meningitis associated with HIV infection.

## Conclusion

Although proper treatment was administered, the mortality in invasive fungal infections remains high, given the fact that they generally appear in already marred or severely immunosuppressed patients.

